# Case Report: Lurasidone-Induced Type 2 Brugada Pattern in a Pediatric Patient

**DOI:** 10.5811/cpcem.1573

**Published:** 2025-05-28

**Authors:** Ethan Start, Aldrin Enabore

**Affiliations:** * University of Florida – Jacksonville, Department of Emergency Medicine, Jacksonville, Florida; † Prisma Health - Upstate, Department of Emergency Medicine, Greenville, South Carolina

**Keywords:** Brugada, pediatrics, case report, lurasidone, anti-psychotic

## Abstract

**Introduction:**

Brugada syndrome, a cardiac channelopathy, manifests with ventricular arrhythmia. Diagnosis relies on a type 1 Brugada electrocardiogram (ECG) pattern, while type 2 and type 3 patterns may necessitate electrophysiologic testing to uncover an underlying type 1 Brugada pattern. Differentiation between these patterns is important, as type 1 patterns pose a significantly greater risk of arrhythmia relative to types 2 and 3 counterparts.

**Case Report:**

A 14-year-old male with autism presented after a syncopal episode following a lurasidone dosage increase. His ECG revealed a type 2 Brugada pattern. He was monitored overnight in the pediatric intensive care unit, where he remained asymptomatic. After being discharged with a Holter monitor, a quaternary hospital’s procainamide challenge test weeks later contradicted an official diagnosis of Brugada syndrome, as dictated by elucidation of a type 1 Brugada pattern. After reverting to the initial lurasidone dose, a follow-up ECG after two months showed no Brugada pattern.

**Conclusion:**

In syncope cases, an ECG is crucial for identifying arrhythmogenic causes, including Brugada syndrome. This case highlights an ECG suggestive of Brugada syndrome with negative pharmacological tests and resolution post-discontinuation of the offending agent. Emergency physicians should be vigilant for Brugada and long QT syndromes in patients on antipsychotic medications.

## INTRODUCTION

**Image 1:**
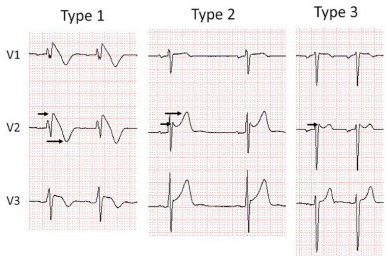
Type 1, 2, 3 Brugada electrocardiogram patterns (left to right). Only type 1 is diagnostic of Brugada syndrome. Type 1 has a coved-shaped ST-segment elevation greater than 2 millimeters (mm) (short arrow), followed by a negative T-wave (long arrow). Type 2 has a saddleback ST-segment elevation greater than 2 mm (short arrow), followed by a positive T-wave (long arrow). Type 3 can resemble either morphology but with less than 2 mm of ST-segment elevation (arrow). Published from the 2016 J-waves syndromes expert consensus conference report.^3^ Reprinted with permission.

Brugada syndrome, an autosomal dominant heart disorder with variable expression, poses a risk of ventricular arrhythmia and sudden cardiac death in individuals with structurally normal hearts, especially among the young. Three distinct Brugada electrocardiogram (ECG) patterns, illustrated in Image 1, have been identified. Of these, only type 1 is considered potentially diagnostic for Brugada syndrome. Type 1 patterns pose a 0.4% yearly risk of arrhythmia, while type 2 and 3 patterns pose a risk at a lesser rate of 0.03%.^1^ Due to the increased risk in patients with definitive type 1 patterns either spontaneously or provocatively with electrophysiologic testing, type 2 and 3 patterns necessitate additional workup to uncover an underlying type 1 Brugada pattern.

The type 1 Brugada pattern is characterized by a coved elevation of at least 2 millimeters (mm) in the ST-segment and a negative T-wave in leads V1–V3. In contrast, the type 2 ECG pattern exhibits a “saddleback” shape, featuring a gradually descending ST-segment elevation greater than 2 mm followed by a positive T-wave. Lastly, type 3 can manifest with either morphology, albeit with less than 2 mm of ST-segment elevation.^2^

Lurasidone hydrochloride is an atypical antipsychotic primarily used as a mood stabilizer in those with behavioral and psychiatric disorders. Common side effects of lurasidone include hypertriglyceridemia, hypercholesterolemia, hyperglycemia, nausea, and extrapyramidal reactions.^4^ Notably, there is no documented association between lurasidone and Brugada syndrome.

## CASE REPORT

A 14-year-old male previously diagnosed with autism, who was taking lurasidone as a behavioral suppressant, presented to the emergency department (ED) after a reported syncopal episode in the bathroom. The patient, along with his mother, denied any previous history of syncopal episodes. In the week leading up to the incident, the patient’s pediatrician had escalated his lurasidone dosage from 20 milligrams (mg) to 40 mg daily due to increased behavioral challenges at school. Aside from this adjustment, the patient reported no recent illnesses or heightened stressors and denied any history of smoking, alcohol use, or illicit drug consumption.

In the ED, his vital signs were as follows: temperature 98.1 °F (36.7 °Celsius), heart rate 70–90 beats per minute, respiratory rate 16–20 breaths per minute, and blood pressure 138/71 millimeters of mercury. Orthostatic vitals were unremarkable for any change in heart rate or blood pressure. Physical examination revealed an alert and oriented Black male in no acute distress. There were no findings concerning for significant trauma or clinical dehydration. He was without any respiratory distress, with lungs clear to auscultation bilaterally. On cardiac auscultation, there was a regular rate and rhythm with no appreciable murmurs. His abdomen was soft, non-tender, and non-distended. He had an appropriate range of all his extremities and ambulated without difficulty. He had an appropriate mood and affect, had no neurological deficits, and was overall asymptomatic.

*CPC-EM Capsule*

What do we already know about this clinical entity?

*Brugada syndrome is a channelopathy associated with electrocardiogram (ECG) abnormalities and sudden cardiac death. Some medications may unmask it*.

What makes this presentation of disease reportable?

*This presentation is reportable because lurasidone has not been previously associated with Brugada patterns*.

What is the major learning point?

*Lurasidone and many other antipsychotics can cause ECG abnormalities and may unmask Brugada patterns*.

How might this improve emergency medicine practice?

*Physicians should be vigilant for Brugada patterns and other ECG findings in syncope patients, especially those on multiple psychotropic medications*.

**Image 2:**
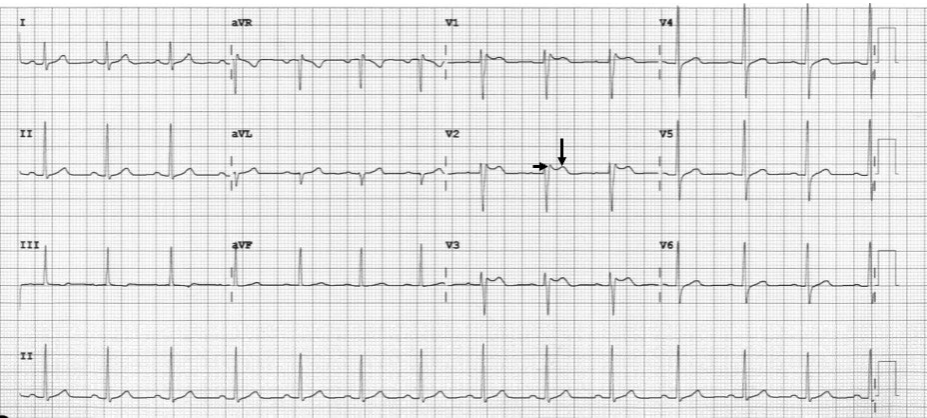
The patient’s initial electrocardiogram reveals a type 2 Brugada pattern. A saddleback ST-segment elevation of 2 millimeters is visible in leads V2 and V3 (short arrow), followed by a positive T-wave (long arrow).

In the ED, an ECG revealed a Type 2 Brugada pattern (Image 2). No prior documented ECG was available for comparison. Following the exclusion of other correctable causes of syncope, pediatric cardiology was consulted, and the attending physician concurred with the diagnosis of a type 2 Brugada pattern. Subsequently, the patient was admitted to the pediatric intensive care unit for continuous telemetry and observation. Throughout the night, the patient remained asymptomatic, and the ECG continued to display the type 2 Brugada pattern the following morning. Cardiology devised a treatment plan consisting of a 24-hour Holter monitor, an outpatient procainamide challenge test at a quaternary-care center, and a reduction of the patient’s lurasidone medication to 20 mg.

Several days later, the patient followed up with his pediatrician for re-evaluation and Holter monitor interrogation. The patient’s initial ECG revealed a type 2 Brugada pattern. A saddleback, 2 mm ST-segment elevation was observed in leads V2 and V3, followed by a positive T-wave. Per the cardiology report:

[T]here is minimal heart rate variability, and only variable results include one episode of sinus tachycardia, and two isolated premature beats likely supraventricular ectopic beats. There is no evidence of tachyarrhythmias or conduction abnormalities or sinus pauses.”

The patient was awaiting a procainamide study scheduled for one week later at a quaternary-care hospital, which was ultimately negative. Throughout this period, the patient remained asymptomatic and exhibited controlled behavior on his initial dose of lurasidone 20 mg.

**Image 3:**
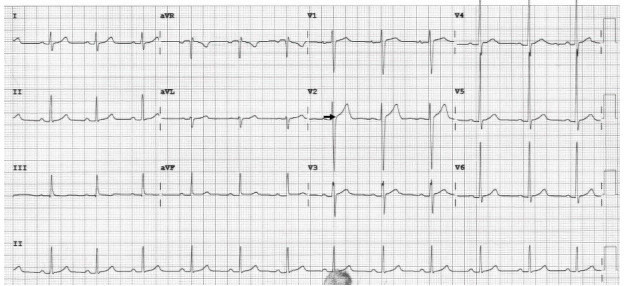
The subsequent electrocardiogram demonstrates the resolution of the Brugada pattern in this patient. Leads V2 and V3 no longer exhibit the coved or saddleback ST-segment elevation (arrow).

One month later, the patient followed up with his primary pediatric cardiologist. His mood was stable on lurasidone 20 mg, and a repeat ECG showed sinus rhythm without any findings suggesting a Brugada pattern (Image 3). During a subsequent phone call with the patient’s family, it was noted that there were no further episodes of syncope, and no planned follow-up appointments with pediatric cardiology were required. There were no additional ED visits.

## DISCUSSION

Brugada syndrome, an autosomal dominant heart disorder with variable expression, poses a risk of ventricular arrhythmia and sudden cardiac death, particularly in young individuals with structurally normal hearts. Men face an elevated risk, and the average age of cardiac arrest is approximately 45 years. Symptoms typically manifest between 20–65 years of age and, although rare, there are documented cases of sudden death due to Brugada syndrome in children.^4^,^5^ The primary therapeutic approach involves implantable cardioverter-defibrillator (ICD) placement. In some cases, patients may receive supplemental quinidine or amiodarone therapy either as a bridge to ICD or to diminish the frequency of ICD shocks.^4^,^5^

Diagnosis of Brugada syndrome hinges on the presence of the type 1 Brugada ECG pattern. According to the 2013 consensus statement by the Heart Rhythm Society, European Heart Rhythm Association, and Asia Pacific Heart Rhythm Society, a definitive diagnosis occurs when a type 1 Brugada ECG pattern is observed spontaneously or following provocative drug testing. For symptomatic patients presenting with a type 2 or type 3 pattern, provocative drug testing with a sodium channel blocker is indicated.^3^ In patients with type 1 Brugada ECG pattern, workup to exclude structural abnormalities may be indicated, as Brugada patterns have been observed as precursor findings of arrhythmogenic right ventricular cardiomyopathy.^2^

Nineteen genes encoding sodium, calcium, and potassium channels have been associated with Brugada syndrome. The most commonly mutated gene is the sodium voltage-gated alpha subunit 5 (seen in ~20–30% of patients). These mutations produce a reduced inward sodium or calcium current or an increased outward potassium current.^4^ These mutations result in both normal and abnormal channels within the epicardium. This results in adjacent myocytes with different refractory periods hypothesized to produce sustained arrhythmias via re-entry during repolarization or abnormal conduction during depolarization.^3^,^4^

Several classes of medications have been shown to induce the Brugada pattern. Sodium channel-blocking medications, such as procainamide, ajmaline, or flecainide, can unmask the Brugada pattern and are sometimes intentionally administered for diagnostic purposes.^5^ The BrugadaDrugs.org registry contains a collection of case reports and mechanistic studies of medications precipitating a Brugada pattern. Per this registry, tricyclic antidepressants, several selective serotonin reuptake inhibitors, several typical antipsychotics, lamotrigine, lithium, and propofol have been listed as drugs that can induce the Brugada ECG pattern. Therefore, caution is recommended in administering these drugs to individuals with Brugada syndrome.^6^

To date, few studies have demonstrated Brugada syndrome due to atypical antipsychotics.^7^ Three case reports have documented the emergence of a type 1 Brugada pattern following clozapine administration, with resolution upon discontinuation.^8^–^10^ Another case involved a 25-year-old male, treated with risperidone for schizophrenia, who developed a type 1 Brugada pattern that spontaneously resolved after discontinuation.^11^ The typical antipsychotics loxapine, trifluoperazine, perphenazine, and thioridazine have also been associated with Brugada syndrome due to the blockade of fast sodium channels.^3^ Intriguingly, patients undergoing treatment for schizophrenia exhibit a higher prevalence of Brugada syndrome; however, whether this is linked to antipsychotic side effects or a genetically related channelopathy remains unknown.^12^ Antipsychotic therapy more commonly is associated with other causes of sudden cardiac death, particularly QT prolongation and subsequent torsades de pointes.^3^

The prognosis of Brugada syndrome is highly variable based on initial presentation. In a study of 1,029 patients diagnosed with Brugada syndrome by type 1 ECG, patients presenting in cardiac arrest had a 35% incidence of ventricular tachyarrhythmia at four years. Six percent of Brugada syndrome patients presenting with syncope had an arrhythmic event at four years.^13^ This massive difference in mortality is likely due to difficulty distinguishing arrhythmic syncope from vasovagal syncope in this cohort. A separate longitudinal study of 1,149 patients demonstrated a 0.4% yearly risk of arrhythmia with spontaneous type 1 Brugada patterns relative to those with provocatively induced type 1 patterns, who carry a significantly lower risk of 0.03%.^1^ In contrast, type 2 and type 3 Brugada patterns that do not convert to a type 1 pattern with electrophysiologic testing are considered non-diagnostic of Brugada syndrome. In a longitudinal study of 18 Finnish patients with type 2 and type 3 ECG patterns, no life-threatening ventricular arrhythmias occurred during a follow-up period of 10–21 years.^14^

## CONCLUSION

In all patients presenting with syncope, an ECG is warranted to identify underlying arrhythmogenic etiologies, including Brugada syndrome. Patients with Brugada syndrome, as diagnosed by a type 1 ECG, are treated with an implantable cardioverter-defibrillator. In contrast, those with lower ventricular arrhythmia risk type 2 and type 3 ECG patterns require additional electrophysiological workup. This case illustrates a transient mimic of a Brugada pattern, characterized by negative results in pharmacological induction tests and ECG resolution after discontinuing the initial causative agent.
